# The Role of EEG-fMRI in Studying Cognitive Network Alterations in Epilepsy

**DOI:** 10.3389/fneur.2019.01033

**Published:** 2019-09-24

**Authors:** Elhum A. Shamshiri, Laurent Sheybani, Serge Vulliemoz

**Affiliations:** ^1^EEG and Epilepsy Unit, Neurology Department, University Hospitals and Faculty of Medicine of Geneva, Geneva, Switzerland; ^2^Neurology Clinic, University Hospitals and Faculty of Medicine of Geneva, Geneva, Switzerland

**Keywords:** EEG-fMRI, epilepsy, review, neuroimaging, interictal epileptiform discharge

## Abstract

Brain functions do not arise from isolated brain regions, but from interactions in widespread networks necessary for both normal and pathological conditions. These Intrinsic Connectivity Networks (ICNs) support cognitive processes such as language, memory, or executive functions, but can be disrupted by epileptic activity. Simultaneous EEG-fMRI can help explore the hemodynamic changes associated with focal or generalized epileptic discharges, thus providing information about both transient and non-transient impairment of cognitive networks related to spatio-temporal overlap with epileptic activity. In the following review, we discuss the importance of interictal discharges and their impact on cognition in different epilepsy syndromes. We explore the cognitive impact of interictal activity in both animal models and human connectivity networks in order to confirm that this effect could have a possible clinical impact for prescribing medication and characterizing post-surgical outcome. Future work is needed to further investigate electrophysiological changes, such as amplitude/latency of single evoked responses or spontaneous epileptic activity in either scalp or intracranial EEG and determine its relative change in hemodynamic response with subsequent network modifications.

## Introduction

Epilepsy cannot be reduced solely to the dysfunction of the seizure onset zone (SOZ), as more widespread abnormalities can be seen, resulting in heterogeneous deficits across cognitive domains (1–6). This supports the view that epilepsy is a network disease associated with complex cognitive deficits (7–11). While these cognitive deficiencies are increasingly recognized as important co-morbidities of epileptic disorders, they are still insufficiently understood and investigated. These deficits can also affect cortical regions that are remote from the epileptogenic zone. For instance, patients with temporal lobe epilepsy can suffer from frontal lobe dysfunction (executive functions) (12, 13). Conversely, patients with frontal lobe epilepsy can suffer from medial temporal lobe dysfunction (memory encoding) (14).

### Epileptic Activity Can Dynamically Affect Cognition

Different hypotheses have tried to explain these deficits. A disruptive role of interictal epileptic discharges (IEDs) during ongoing physiological activity has been shown even if these discharges do not result in clinical signs of a seizure; the occurrence of IEDs can therefore be related to transient cognitive impairment (15–18). Previous studies based on intracranial EEG have investigated how epileptic activity can alter normal cognitive processing through large-scale network disruption (16–18); however, due to the low spatial sampling of electrophysiological recordings, it is often challenging to map these networks without prior assumptions on the relevant brain regions to be recorded. Although intracranial EEG has high temporal and spatial resolutions, it has a low spatial sampling, thus preventing this tool to be used alone to investigate large-scale networks.

### Interactions Between Epileptic Activity and Cognitive Networks

Cognition engages large-scale brain networks (19–21). Resting-state fMRI (rsfMRI) investigates synchronous activity between regions in the absence of an explicit task and can be subdivided into Intrinsic Connectivity Networks (ICNs) (22). The spatial organization of ICNs has been consistent with relevant cognitive tasks, however with subtle variations (23). As such, previous studies have implied that cognitive networks remain dynamically active even during periods of rest (24, 25). The effect of interictal activity could explain part of the nature of cognitive dysfunction in patients with epilepsy. So far, studies have mostly focused on the cognitive disturbances associated with the occurrence of IEDs (15–18). However, the interactions between detailed spatio-temporal aspects of epileptic activity and changes in ICNs and task-related cognitive networks have not been greatly explored. Therefore, the current review will discuss the current applications of EEG-fMRI in relation to cognition in both human and animal studies.

### EEG-fMRI

The simultaneous recording of EEG and fMRI allows for data acquisition with high spatio-temporal resolution, thereby making it possible to map hemodynamic changes related to interictal epileptic activity (26, 27). EEG-fMRI is classically used to estimate the localization of the epileptogenic zone in the context of pre-surgical investigation of epilepsies (28–31), and only a few studies have used EEG-fMRI to investigate the direct effect of epileptic activity on cognition (22, 32).

## Methods

**Figure 1:**
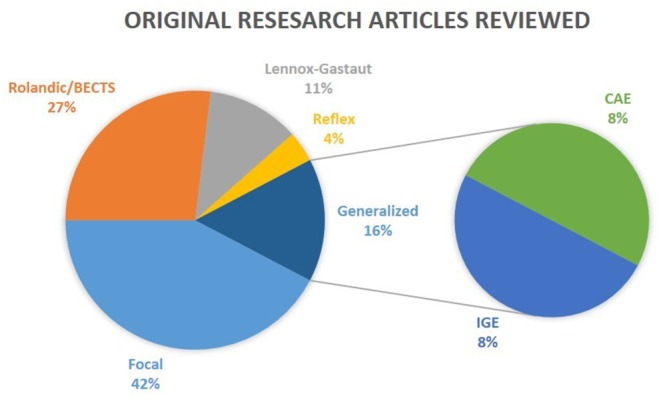
Epilepsy types reviewed. The results of the review on clinical studies are separated into five distinct categories: Rolandic/BECTS, Lennox-Gastaut, Reflex, Focal, and Generalized. CAE and IGE patients are considered subgroups of Generalized epilepsy. Reviews were not taken into consideration in this illustration.

For this review, we performed a comprehensive literature search on the Medline PubMed database of all original research articles to date (July 2019) within the last 5 years (see Figure 1) with the keywords: (1) “epilepsy” AND “cognitive OR cognition” AND “EEG-fMRI”, (2) “epilepsy” AND “cognitive OR cognition” AND “EEG AND fMRI AND simultaneous.” However, due to the restrictive parameters, we only received one paper as a result in animal studies; therefore the parameters were extended to become more permissive by excluding the “cognitive OR cognition” criteria and expanding the timeline. Articles were excluded from the review if they were case studies or not in English. Some of the resulting papers (see Table 1) were methods-based, and were therefore summarized in the review, but not explained in detail as the purpose was to explore the role of EEG-fMRI in cognition. In the following sections, we discuss the role of EEG-fMRI in investigating the interaction between epileptic discharges and cognitive networks.

**Table 1:** Epilepsy types reviewed.

	**Type of epilepsy**	**Primary question**	**General result/observation**	**References**	**Statistical analysis**	**# of SUBJECTS**	**Age range**
**(A) CLINICAL STUDIES**							
1	Focal epilepsy (mTLE only)	What are the changes in the DMN, SN, and DAN networks in relation to the onset of interictal spikes?	Decreased synchronization of FC prior to the onset of interictal spikes	(33)	Functional connectivity	Patients = 15, controls = 15	Adults
2	Focal epilepsy	What is the value of IED-related BOLD maps in terms of pre-surgical planning?	Overlapping of IED-related BOLD maps with surgical resection is a marker of good prognosis	(34)	IED-related map and comparison with surgical resection	Patients = 30	Mixed: children and adults
3	Focal/Generalized	Can we account for the behavior of epileptic generators when no spikes are visible? And will this improve localization?	Yes, and it improves upon traditional spike-based analysis	(35)	GLM	Patients = 20, controls = 20	Mixed: children and adults
4	Focal epilepsy (mTLE only)	What are the changes in FC prior to spike onset in mTLE?	Significant loss of synchronization between bilateral hippocampi during the pre-spike periods	(36)	Functional connectivity	Patients = 15, controls = 15	Adults
5	Focal epilepsy	Can solely fMRI-driven results be used to localize the focus?	Yes, and it could be useful for EEG-negative patients	(37)	ICA and a cascade of classifiers	Patients training set = 12, patients test set = 18, controls = 13	Adults
6	Focal epilepsy	Is there an identifiable epileptic network outside the occurrence of IEDs?	The connectivity of the epileptic network remains high after removal of the IED contribution	(38)	Comparison of the IED-related network as identified by fMRI (ICA with best overlap with EEG-driven network) and the one identified by EEG	Patients = 10	Mixed: children and adults
7	Focal epilepsy	Does a new fast fMRI sequence (MREG) increase sensitivity to detect IED BOLD-related changes?	MREG increases sensitivity in detecting negative BOLD responses of IEDs in the DMN	(39)	GLM	Patients = 15	Mixed: children and adults
8	Focal epilepsy	Comparison of functional networks between patients with focal epilepsy and controls	Patients show higher local connectivity and decreased long-range connections; Epochs with and without IEDs do not change significantly	(40)	Functional connectivity maps	Patients = 23, controls = 63	Mixed: children and adults
9	Focal/Generalized	Review	Simultaneous EEG-fMRI can help delineate epileptic foci and propagation pathways using rsfMRI	(41)	N/A	N/A	N/A
10	Focal/Generalized	Review	Simultaneous EEG-fMRI improves our understanding of the electrophysiological correlates of epileptic/BOLD activity	(42)	N/A	N/A	N/A
11	Focal epilepsy	Are early BOLD responses in epilepsy patients a result of “temporal bleeding”	The HRF is affected by “temporal bleeding”? ±3 sec; authors recommend using a HRF at−6 sec to avoid “temporal bleeding”	(43)	GLM	Patients = 7, controls = 6	Adults
12	Focal epilepsy	Can task-induced HFAs be seen in simultaneous iEEG-fMRI?	HFAs can be reliably seen in iEEG-fMRI	(44)	Multi/single-trial analysis	Patients = 3	Adults
**(A) CLINICAL STUDIES**							
13	Focal epilepsy	What is the impact of interictal IEDs on ICNs (ECN and VN) in pediatric patients?	When IEDs are controlled for, ICNs are not different in patients vs. controls	(22)	Functional connectivity	Patients = 27, controls = 17	Children
14	Focal epilepsy (TLE only)	What are the real-time effects of IEDs on hippocampus and amygdala FC?	IEDs in the left hemisphere disconnected the left hippocampus and the DMN	(45)	Dynamic FC	Patients = 21	Mixed: children and adults
15	Focal epilepsy	Is EEG-fMRI accurate in detecting the ictal onset zone at varying statistical thresholds?	Increased sensitivity and specificity was achieved using a specific threshold	(46)	GLM and ROC curves	Patients = 21, controls = 21	Adults
16	Focal/Generalized	Review	EEG-fMRI can be used to localizing epileptic networks	(47)	N/A	N/A	N/A
17	Focal epilepsy (mTLE only)	Can amplitude of low frequency fluctuations (ALFF) and FCD be used for localization?	Increased ALFF is in mTLE structures and decreased FC attributed to desychronization between mTLE structures and the whole brain	(48)	ALFF and FCD	L mTLE patients = 26, R mTLE patients = 21	Adults
18	BECTS	How do IEDs affect ICNs (AN, BGN, DAN, DMN, SMN)?	Patients with IEDs show decreased FC in the DMN	(49)	Functional connectivity	Patients = 43, controls = 28	Children
19	BECTS	What are the dynamic changes seen in FC of BECTS patients?	Patients showed decreased dynamic FC in the orbital frontal cortex, ACC, and striatum; furthermore, both active and chronic effects of epilepsy contribute to altered dynamics of FC	(50)	Dynamic FC	Patients = 45, controls = 28	Children
20	BECTS	How does epileptic activity interfere with whole-brain networks?	Functional defects in brain networks contribute to patient symptomatology (i.e.: decreased nodal centralities in areas related to linguistics and attention control)	(51)	Functional connectivity and graph theory metrics	Patients = 73, controls = 73	Children
21	BECTS	Do BECTS patients with ADHD show specific network changes in comparison to patients without ADHD/healthy controls?	BECTS patients with ADHD show decreases in FC in the DAN in comparison to BECTS patients without ADHD/controls	(52)	Functional connectivity	Patients with ADHD = 15, patients without ADHD = 15, controls = 15	Children
22	BECTS	What are the real-time effects of spikes on cognitive function (i.e.,: language and behavior)	Interictal CTS disrupts networks involved in cognition (positive correlation between bilateral BECTS areas and left IFG/Broca's area)	(53)	Dynamic FC	Patients (medication-naïve) = 22	Children
23	BECTS	What is the effect of Levetiracetam on activations/deactivations and CTS?	Overall decreased activation (in higher cognition networks) in the medicated group compared to the drug-naïve patients	(54)	GLM	Medicated patients = 20, drug-naïve patients = 20	Children
24	BECTS	Can network abnormalities be used to differentiate between patients without IEDs and controls?	Patients without IEDs can be distinguished from controls	(55)	Amplitude of low frequency fluctuations and multivariate pattern classification	Patients with IEDs = 20, patients without IEDs = 23, controls = 28	Children
**(A) CLINICAL STUDIES**							
25	Lennox-Gastaut	Review	Epileptic activity in LGS can be seen in large scale networks such as attention default mode networks and can be categorized as a “secondary network epilepsy”	(56)	N/A	N/A	N/A
26	Lennox-Gastaut	Are the affects of LGS on cognitive networks persistently abnormal?	Abnormal connectivity was present during periods with/without IEDs	(57)	Functional connectivity	Patients = 15, controls = 17	Mixed: children and adults
27	Lennox-Gastaut	How does the FC change in a LGS patient with good post-surgical outcome?	Increased small-worldness, stronger connectivity subcortically, and greater within-network integration (between-network segregation)	(58)	Functional connectivity and graph theory metrics	Patient with good post-surgical outcome = 1, patients with no surgery = 9	Children
28	Lennox-Gastaut	What are the brain regions underlying interictal generalized proxysmal fast activity (GPFA)?	GPFA propagates from the prefrontal cortex to the brainstem via corticoreticular pathways; this network is present in both children and adults	(59)	Event-related analysis and DCM	Patients under anesthesia = 10, patients without anesthesia = 15	Mixed: children and adults
29	Reflex epilepsy	What are the regions associated with the initiation of seizures in reflex epilepsy?	Different networks show changes related to a specific type of reflex epilepsy (startle myoclonus, eating, and hot water)	(60)	GLM	Patients = 3	Mixed: children and adults
30	IGE	What regions terminate absence seizures?	Lateral prefrontal cortex involved at GSWD termination	(61)	Event-related analysis	Patients = 18	Mixed: children and adults
31	EMA, IGE	What are the structural/functional changes in EMA and IGE patients with epileptic activity triggered by eye closure?	Functional changes show increased activity in visual cortex, posterior thalamus, and motor control; structural changes include gray matter increases in visual cortex and decreases in frontal eye fields	(62)	Random-effects analysis and VBM	EMA patients = 15, IGE patients = 14, controls = 16	Mixed: children and adults
32	CAE	How do network properties change during seizure onset and offset in the DMN and thalamus networks?	There is an anti-correlation between the thalamus and DMN, which gradually decreases after seizure onset	(63)	Dynamic FC and graph theory metrics	Patients = 11	Children
33	CAE	How do GSWDs impact different ICNs and cognitive processes?	ICNs associated with higher-order cognitive processes (DMN, CEN, DAN, SN) had decreased connectivity while perceptive/motor processes were spared; ICNs showed different temporal responses to GSWDs illustrating a hierarchy	(48)	GLM and ICA	Patients = 16	Children
34	Genetic epilepsy (ring chromosome 20)	Review	Patients have both interictal and ictal disruptions in basal ganglia-prefrontal networks	(64)	N/A	N/A	N/A
**(B) ANIMAL STUDIES**							
1	No epileptic disorder	Proof of principle study for studying combined optogenetic stimulation, electrophysiology, and fMRI acquisition	Optogenetic stimulation elicits large-scale BOLD activity network, not restricted to the stimulated site	(65)	fMRI, LFP measurement, frequency analysis	13 rats (see paper for # of animals per experiment)	N/A
2	Pilocarpine- and electrically-induced limbic seizures	What is the nature of ictal neocortical slow-waves during limbic seizures?	Neocortical slow-wave represent decreased activity in the neocortex, not seizure propagation	(66)	LFP identification of seizure and BOLD-activity based map related to seizures	62 rats	N/A
3	No epileptic disorder	What is the neuronal activity underlying resting state functional connectivity?	Differential contribution of LFP frequency bands in BOLD signal	(67)	LFP-BOLD power-power correlation and phase-amplitude coupling	29 rats	N/A
4	No epileptic disorder	Is combined optogenetic-fMRI reliable to study large-scale network?	Methodological paper making optogenetic-fMRI a suitable method to study large-scale networks	(68)	Large-scale BOLD activity (see paper for details)	3–8 rats per experiment	N(A
5	Electrically-induced focal seizures	What is the biological substrate of decreased consciousness in focal seizures?	Decreased activity of subcortical arousal systems leads to decreased cortical function	(69)	BOLD activity, electrophysiology, and amperometry-based neurotransmitter measures	Total of 138 rats (see paper for specific experiments)	N/A
6	Animal model of absence epilepsy and bicuculline-induced GTCS	What is the BOLD network associated with SWD and GTCS of generalized epilepsy?	Increase BOLD activity in somatosensory cortex and thalamus, decrease in occipital cortex	(70)	Large-scale BOLD activity related to epileptic activity	16 rats	N/A
7	No epileptic disorder	What is the neuronal activity underlying the BOLD activity?	BOLD fluctuation correlate with power of γ-range LFP activity, more than with AP frequency	(71)	Analyses of BOLD-LFP correlation under visual stimulation	5 cats	N/A
8	GHB animal model of absence epilepsy	What is the regional BOLD activity during absence seizures?	(i) BOLD increase in thalamus (ii) BOLD decrease in motor and temporal cortex (iii) Heterogeneous BOLD response in parietal cortex	(72)	Comparing alternating periods of rest and induced absence seizures via GLM	8 rats	N/A
9	WAG/Rij rat model of spontaneous absence seizures	What is the regional BOLD activity during absence seizures?	(i) BOLD increase in thalamus (ii) Widespread cortical increase (temporal, parietal) (iii) No negative BOLD identified	(73)	Comparing alternating periods of rest and induced absence seizures via GLM	10 rats	N/A
**(B) ANIMAL STUDIES**							
10	GBL non-human primate model of absence epilepsy	Development of a non-human primate model of absence epilepsy to study the regional BOLD activation during absence seizure	(i) BOLD increase in widespread cortical regions (pre-/post-central, frontal, and temporal cortices, thalamus) (ii) No negative BOLD identified	(74)	Comparing alternating periods of rest and induced absence seizures via GLM	6 marmoset monkeys	N/A

*Section A refers to the clinical studies that resulted from the search criteria. The last 5 years produced 34 papers from 2014 to 2019 (five of which were reviews and are written in red). Section B displays the search for animal studies, which went beyond the 5 years criterion due to otherwise limited results and produced 10 papers. Methodological papers that did not recruit patients/animals with epilepsy are written in blue. The table is organized by alphabetical order (of the first author). ACC, Anterior Cingulate Cortex; ALFF, Amplitude of Low Frequency Fluctuations; AN, Auditory Network; AP, Action Potential; BECTS, Benign Epilepsy with Centro-Temporal Spikes; BGN, Basal Ganglia Network; CAE, Childhood Absence Epilepsy; CEN, Central Executive Network; CTS, Centrotemporal Spikes; DAN, Dorsal Attention Network; DMN, Default Mode Network; ECN, Executive Control Network; EMA, Eyelid Myoclonus with Absences; FCD, Functional Connectivity Density; GBL, γ-ButyroLactone; GHB, γ-HydroxyButyric acid; GLM, General Linear Model; GPFA, Generalized Paroxysmal Fast Activity; GSWD, Generalized Spike-Wave Discharges; GTCS, Generalized Tonic-Clonic Seizure; HFA, High Frequency Activity; HRF, Hemodynamic Response Function; ICA, Independent Component Analysis; icEEG, intracranial EEG; IED, Interictal Epileptiform Discharge; IFG, Inferior Frontal Gyrus; IGE, Idiopathic Generalized Epilepsy; LFP, Local Field Potential; MREG, Magnetic Resonance Encephalography; mTLE, mesial Temporal Lobe Epilepsy; rsfMRI, resting state functional Magnetic Resonance Imaging; SN, Salience Network; SWD, Slow-Wave Discharge; VBM, Voxel-Based Morphometry; VN, Visual Network; WM, Working Memory*.

### EEG-fMRI in Animal Models of Epilepsy

The use of combined EEG-fMRI in animal models, and in particular animal models of epilepsy, comes with two major benefits: first, it allows us to control for more parameters than in human research, thus providing more insights into the biological substrates of the BOLD signal, as illustrated by studies using optogenetic tools (65). Second, it gives access to the epileptic network (10, 11, 75), as it offers the opportunity to sample multiple brain regions related to the activity of the epileptic focus, with much higher spatial and temporal resolution in comparison to studies in humans (76).

BOLD signal analysis can highlight the network recruited during epileptic seizures. Different studies on animal models of generalized seizures (70, 72–74) have shown that the increase in BOLD activity is heterogeneous, and involves specifically thalamo-cortical circuits. These results are in line with the hypothesis that generalized seizures actually represent rapidly-propagating seizures with bilateral onset (77). Thus, fMRI signal can be used to map the network related to one particular “pre-identified” neural activity.

The inverse approach, i.e., to use BOLD signal to identify regions of interest and then guide electrophysiological recordings, is also a powerful tool, as shown in an elegant study in a rat model of temporal lobe epilepsy (69). In this study, the authors investigated the mechanisms of loss of consciousness using EEG-fMRI together with choline amperometry recordings. In short, they found that during focal limbic seizures, BOLD signal increases in the hippocampus [as expected (66)] and also decreases in cortical areas. This result was associated with a decreased firing of cholinergic neurons, but not non-cholinergic neurons, in the subcortical arousal system of the brainstem (69). This could explain, at least in part, the alteration of arousal during focal seizure. Very brief or partial arousal impairment could play an important role in transient cognitive impairments. Therefore, BOLD-guided electrophysiology provides a complementary tool to investigate the perturbation of brain networks during seizures. Aside from consciousness, EEG-fMRI studies of cognition in animals have remained scarce thus far (67, 71).

### EEG-fMRI in the Study of Cognition in Humans

Previous studies have commented on the relationship between cognition and ICNs extracted from traditional resting state fMRI, especially in relation to patients with epilepsy (78–80). ICNs can be ascribed to specific functions, such as self-awareness, attention, cognitive control, or perceptions such as visual, auditory, or motor (81–83). There is some spatial overlap between these networks in both patients and healthy controls; however the abnormal modulation of activity between these networks can be indicative of a patient's clinical syndrome.

Over the last 5 years there has been a substantial increase in the use of EEG-fMRI, especially for pre-surgical evaluations for patients with epilepsy (7, 35, 37, 41, 46, 47). However, the effects of IEDs on cognitive networks were not often explored until recently. Following pioneering work relating IED-correlated decreases in Default Mode Network activity in temporal lobe epilepsy (84) and generalized epilepsy (85), recent works have shown the possible impact of interictal activity on several ICNs in focal epilepsy in adults (33, 36, 45), focal epilepsy in children (22), children with idiopathic focal epilepsy [Benign Epilepsy with Centro-temporal Spikes (BECTS)] (53, 55, 86), epileptic encephalopathy (56–59), as well as generalized epilepsies (61, 64), including Childhood Absence Epilepsy (CAE) (87), and even reflex epilepsies (60). The majority of recent EEG-fMRI studies who evaluate the interaction between interictal discharges, ICNs, and their relationship to neuropsychological outcome have been in BECTS patients; these studies found a negative correlation between cognitive functioning and Functional Connectivity (FC). Nevertheless, though patients with epilepsy are a heterogeneous population, all groups show a widespread influence of interictal activity on ICNs; as ICNs have previously been related to cognitive function, this strengthens the notion that IEDs have a definitive impact on cognitive functioning.

### IEDs and Cognitive Performance

There are two ways to study the impact of IEDs on cognitive processing. One is to compare cognitive processing between patients with different IED occurrences (or other IED parameters such as: duration, or periods before vs. after onset of IEDs). Some evidence suggests that IEDs can be a marker of poor cognitive prognosis (88, 89) and their treatment could improve behavior in children (90). IED burden also plays a role, as shown by the fact that a diurnal occurrence of IEDs >10% of EEG duration is correlated with poorer information processing speed, verbal memory and visuo-motor integration in children (91).

Another way to probe the mechanisms through which IEDs perturb cognitive functions is to ask whether or not the occurrence of a single IED can directly affect brain processing. Indeed, IEDs could affect normal cognitive processing through *transient* disruption of brain networks, a paradigm known as *transitory cognitive impairment* (TCI) (92). Aarts et al. (93) showed that the occurrence of IEDs in patients with different kinds of epilepsy affected performance during a cognitive task, and further showed that left-sided IEDs tended to elicit errors in the verbal task and right-sided IEDs in the non-verbal task. Kleen et al. and Ung et al. added a level of complexity by showing that the laterality of the IEDs relative to the epileptic focus determined the existence of abnormal processing. It is interesting to observe that cognitive processing in turn can also modulate IED frequency (17, 94). An increase of temporal IEDs was indeed observed during cognitive tasks involving temporal structures (94), suggesting that increases in physiological activity might also favor the recruitment of local pathological networks. This further entangles the relationship between epileptic and physiological activity.

### IEDs and Cognitive Networks

These studies highlight the fact that consideration of IEDs has to be integrated with network imaging to understand how IEDs affect brain processing. This was investigated in a patient with idiopathic generalized epilepsy using EEG-fMRI during a memory task, which showed that IEDs perturb the brain network recruited by the task (95). Furthermore, recent studies have found that IEDs interfere with whole brain networks (49, 51), and indeed a recent review found a consensus between studies in both BECTS and CAE patients confirming the significant impact of IEDs on FC measurements (96).

If IEDs and sub-clinical features affect ICNs and therefore the underlying cognitive attributes, the next step is to understand when and how these changes occur. To answer the first question, both Burianová et al. (33) and Faizo et al. (36) explored connectivity prior to IED onset in TLE patients to determine the temporal extent at which connectivity is altered. Regardless of the presence of IEDs, both studies showed patients with abnormal connectivity networks. Burianová et al. (33) demonstrated decreases in functional connectivity (FC) in prefrontal cortices and increases in subcortical areas such as the thalamus (33). However, FC changes were also found prior to IED onset in hippocampal areas (36), thus corroborating the evidence suggesting decreases in FC between the hippocampus and PFC in TLE patients (28). They also found reduced connectivity of the DMN, which occurred prior to IED periods, while reduced connectivity of the salience network occurred during IED periods, relating to behavioral changes in consciousness and attention. Changes in connectivity seen prior to IEDs are particularly interesting as pre-IED hemodynamic changes have also been seen when studying the hemodynamic response function using deconvolution (43, 97, 98). Though the origin of this phenomenon is still unknown, it certainly reflects the existence of pre-IED specific neuronal activity. It would be interesting for future studies to explore the variability of HRF change in this context.

### Transient Effects of IEDs On Epileptic and Cognitive Networks

Differences in connectivity measures remain in the absence of IED activity and this implies a separation between “transient” and “non-transient” effects. This can be seen in both adults and children. The connectivity pattern obtained from IED-correlated fMRI analysis is largely preserved in the absence of IEDs (38, 40).

**Figure 2:**
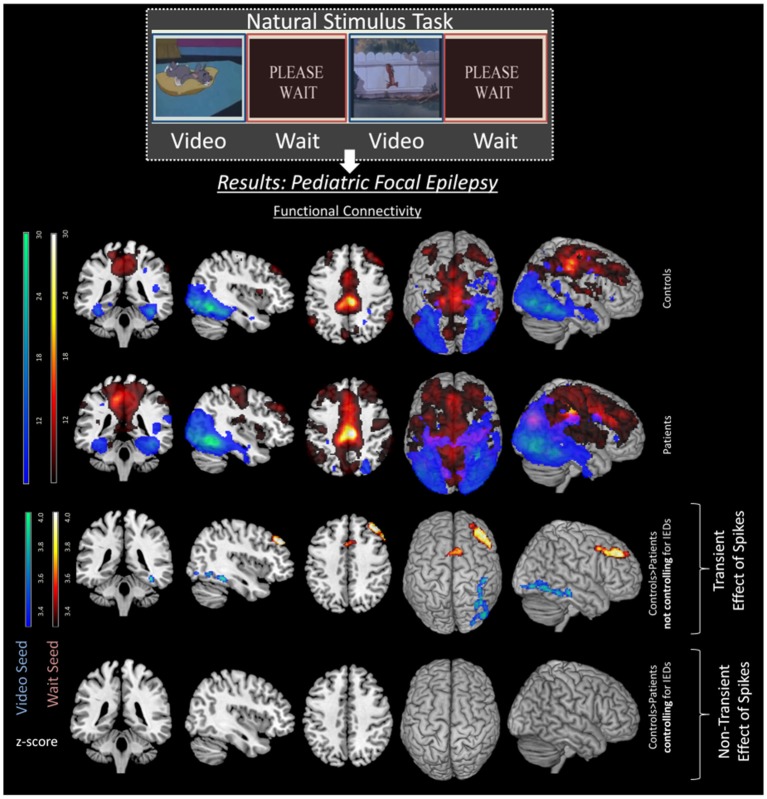
Transient effects of IEDs in pediatric focal epilepsy patients. Image with permission Shamshiri et al. (22) illustrating the effects of spikes on FC networks of a resting state task. Differences between controls (top row) and patients (second row) can be seen in the third row. These differences are including both transient and long-term effects of spikes as spikes are not controlled for in the analysis. However, once the transient effects of spikes are accounted for, the group differences disappear (fourth row), emphasizing the effect of IEDs on ICNs.

Regarding cognition, Shamshiri et al. (22) found connectivity differences in cognitive networks (related to attention) in a group of children with focal epilepsy compared to controls. However, no evidence remained for non-transient differences in network connectivity between patients and controls, after accounting for IED effects (see Figure 2). These results were also consistent with a MEG study in children with focal epilepsy patients by Ibrahim et al. (99), but are inconsistent with those studies mentioned above (33, 36), possibly due to differences between adult and pediatric populations and their respective variability in plasticity and disease duration (99). Instead, for BECTS patients, several studies reported decreases in functional connectivity regardless of the presence of IEDs (50, 51, 86, 100). These patients showed decreased FC in the inferior frontal gyrus, anterior cingulate cortex, and the striatum, which have previously been related to cognitive control (86). This is particularly interesting as patients with BECTS often display behavioral difficulties and language delays (53). However, the effect of medication should also be taken under consideration when determining differences in functional connectivity. Indeed studies in BECTS patients have shown decreased connectivity in higher order functioning cognitive networks of drug naïve patients in comparison to medicated patients (54). The investigation of the difference between transient vs. non-transient changes in connectivity could benefit from simultaneous EEG-fMRI recordings and accounting for the age-related influence on long-term connectivity changes.

### Spatial Considerations of IEDs

It is not only the temporal dynamics of interictal activity that are interesting, but also where these events occur. Indeed the spatial pattern can have an influence on which cognitive domain is predominantly affected. For example, in TLE patients the laterality of IED activity can preferentially damage certain cognitive abilities, such that left temporal IEDs were associated with disconnections to the hippocampus and the Default Mode Network (DMN) while right temporal IEDs were co-activated with the reward-emotion network, which could be involved in forced normalization (a condition in which patients show psychiatric degradation when the IEDs disappear under treatment) (45).

In contrast to local IEDs, such as those seen in TLE patients, generalized (bilateral synchronous) epileptic activity can have a more global effect on ICNs. CAE patients also have widespread GSWD-related decreases found in DMN, DAN, central executive, and salience networks (87). Also, in ring chromosome 20 syndrome, which is a rare and severe form of generalized epilepsy, increases in slow wave rhythm were related to decreases in activity of the DMN and Dorsal Attention Network (DAN) (64). However, the clinical meaning of this slow-wave activity, and whether it supplies transient or long-term effects on cognition, is still under debate. Patients with Lennox-Gastaut syndrome suffer from diffuse cognitive impairment and present widespread, often “generalized” epileptiform activity. In this group, there is no difference in network behavior between fMRI periods affected or unaffected by discharges (101). This pattern is in favor of a more chronic and enduring impairment in this condition, as reflected by the associated encephalopathy. Therefore, generalized epilepsies also show widespread decreases in ICNs especially corresponding to higher order cognitive processes (64, 87).

### Perspectives

The study of IED-related effects on cognitive networks may be difficult in many patients, given the lack of frequent IEDs to model. Other approaches to model pathologic activity using EEG topographies (31, 34) or other EEG features such as decomposition using Independent Component Analysis (102) may offer alternative markers of epileptic activity to correlate with cognitive network alterations.

Simultaneous intracranial EEG and fMRI would allow to better map fMRI network alterations correlated to intracranial pathological EEG activity. Such recordings (103, 104) focused on the mapping of epileptic network (32) and the coupling between neuronal activity and hemodynamic changes, which is related to the fundamental assumptions underlying fMRI studies. These fMRI studies take advantage of the relationship between neuronal activity (mainly post-synaptic potentials) and deoxyhemoglobin concentration (42) to show the focal changes related to the epileptogenic zone, and reveal distant BOLD modulations related to the interictal epileptic network (104). Simultaneous recordings of intracranial EEG and scalp EEG could also uncover new non-invasive markers of epileptic activity that are currently undetectable on scalp EEG but could nevertheless affect cognitive processing. Such markers could be used to refine EEG-fMRI analysis (105, 106).

The possibility to inform fMRI analysis using EEG-derived brain activity offers several perspectives to study the spatio-temporal aspects of cognitive networks, at rest or engaged in specific tasks, in a more selective way than using fMRI, EEG or MEG alone. The characteristics of task-related EEG evoked responses (amplitude, latency) can be included in the fMRI analysis to model and map the network involved in such responses, such as attention and error monitoring (107, 108) and therefore also study interactions with epileptic activity. EEG measures of arousal (e.g., drowsiness or sleep) could also be valuable to study alterations of cognitive networks. Changes in arousal have a significant effect on fMRI connectivity patterns than can even be used to monitor drowsiness during scanning (109). This could be particularly relevant when studying patients with epilepsy vs. controls when drowsiness could show group differences, notably related to drug-induced sedation, sleep deprivation or scanner related anxiety. Antiepileptic drugs affect fMRI brain networks in healthy controls (110) and the effect other drugs, such as donepezil and memantine in the field of dementia, have also been documented (111). This contribution of medication is hard to disentangle from the effect of disease, notably due to the high variability of drug regimes in patient groups and the difficulty to recruit drug naïve patients. EEG markers of medication, such as beta activity or increased drowsiness could be used to try to model this effect in the analysis.

Conversely, fMRI offers the possibility of high spatial resolution to localize cortical and subcortical brain regions at a whole brain scale that are involved in EEG patterns and therefore make it superior in this regard to source localization and connectivity measures based on EEG or MEG alone. Also, taking advantage of the combined high temporal and spatial resolution of EEG and fMRI, EEG connectivity analysis describing directed connections and dynamic aspects (high temporal resolution) could be based on spatial networks revealed by fMRI (whole brain, high spatial resolution) to enhance network characterization.

Future studies could also address the relationship between IEDs and brain rhythms (11, 17), and how this disrupts normal cognitive processing, which are known to rely on specific brain oscillatory activity (19, 20, 112–114).

## Conclusion

Overall, the temporal and spatial effects of epileptic activity and medication can all influence changes in ICNs and cognitive functioning. Although there has been an increase in interest regarding EEG-fMRI and the effects of epileptic activity on ICNs, as reflected by the number of results from our search (see Figure 1, and Table 1), there is still much to learn about how to use this information to understand the long-term impact of interictal activity and cognition and improve the decision making regarding the therapy of patients with epilepsy. Globally, there are differences between focal/non-focal epilepsies, especially in regards to which ICNs or task-related networks are more sensitive to IEDs and how the epileptogenic network influenced the findings. Nevertheless all groups show a widespread influence of interictal activity but also some IED-independent alterations.

## Author Contributions

ES researched, wrote, and reviewed all work pertaining to human subjects. LS researched, wrote, and reviewed all work pertaining to animal models. SV edited all work that was reviewed in this article.

### Conflict of Interest

The authors declare that the research was conducted in the absence of any commercial or financial relationships that could be construed as a potential conflict of interest.
